# Survival rate of primary molar restorations is not influenced by hand mixed or encapsulated GIC: 24 months RCT

**DOI:** 10.1186/s12903-021-01710-0

**Published:** 2021-07-23

**Authors:** Rodolfo de Carvalho Oliveira, Lucila Basto Camargo, Tatiane Fernandes Novaes, Laura Regina Antunes Pontes, Isabel Cristina Olegário, Thais Gimenez, Ana Laura Pássaro, Tamara Kerber Tedesco, Mariana Minatel Braga, Fausto Medeiros Mendes, Daniela Prócida Raggio

**Affiliations:** 1 Department of Orthodontics and Pediatric Dentistry, University of São Paulo, São Paulo, Brazil; 2 Department of Pediatric Dentistry, Paulista University, Campinas, Brazil; 3 Department of Dentistry, Cruzeiro Do Sul University, São Paulo, Brazil; 4 Department of Public & Child Dental Health, Dublin Dental University Hospital, Trinitty College Dublin, Dublin, Ireland; 5 Department of Dentistry, Ibirapuera University, São Paulo, Brazil; 6 School of Dentistry, University of Sao Paulo, Av. Professor Lineu Prestes, 2227, São Paulo, SP 05508-000 Brazil

**Keywords:** Dental restoration, Glass ionomer cement, Randomized Clinical Trials

## Abstract

**Background:**

Glass ionomer cements (GIC) have been considered the top option to restore primary teeth by dentists. The most common supply forms are hand mixed and encapsulated GIC. There is a lack of information about the impact of different GIC supply forms on restoration survival.

**Methods:**

This randomized clinical trial compared the survival rate of occlusal and occlusoproximal restorations in primary molars using two glass ionomer cements versions: hand-mixed (H/M) and encapsulated (ENC) after 24 months. Children aged 3–10 years who presented dentin caries lesions in primary molars were selected at School of Dentistry, University of São Paulo, Brazil. They were randomly assigned to groups: H /M (Fuji IX^®^, GC Europe) or ENC (Equia Fill^®^, GC Europe). The occurrence of restoration failure was evaluated by two blinded and calibrated examiners. The analyses were performed in Stata 13 (StataCorp, USA). To evaluate the primary outcome (restoration survival), we  performed a survival analysis. Additionally an intention to treat (ITT) analysis were done at 24 months of follow-up. Cox Regression with shared frailty was performed to assess association between restoration failure and independent variables (α = 5%).

**Results:**

A total of 324 restorations were performed in 145 children. The survival for H/M group was 58.2% and 60.1% for ENC, with no difference (*p* = 0.738). Occlusoproximal restorations had lower survival rate when compared to occlusal ones (HR = 3.83; *p* < 0.001).

**Conclusions:**

The survival rate in primary molars is not influenced by the different supply forms of GIC. Also, occlusoproximal restorations present reduced performances when compared to occlusal cavities.

***Trial Registration*:**

This randomized clinical trial was registered on ClinicalTrials.Gov on 10/15/2014 under protocol (NCT 02274142).

**Supplementary Information:**

The online version contains supplementary material available at 10.1186/s12903-021-01710-0.

## Background

Glass ionomer cements (GIC) have been considered the top option to restore primary teeth by dentists [1]. Their properties as chemical bonding to enamel and dentin, fluoride release and uptake, thermal expansion coefficient similar to the tooth, and lower sensitivity to humidity than the composite resin (CR) [2–4] favor their choice. Several studies show that GIC restorations have good clinical results both in primary and permanent dentition mainly focused on Atraumatic Restorative Treatment (ART) [5–7]. Therefore, it is essential to achieve the clinical benefit that GIC can provide by understanding the advantages and difficulties offered by the material.

The most common presentation is the hand-mixed GICs (H/M) [5, 7–10], which require correct dispensing and mixing as specified by the manufacturer. Nevertheless, this GIC allow changing the powder-liquid ratio, making it more or less fluid, according to the professional's preference. However, it is not recommended as this could impair the cement's mechanical properties [11] and jeopardize restoration longevity [12–14]. Moreover, incorrect hand-mixing of a GIC could lead to air incorporation into the material matrix and also have an impact on the material properties [14, 15].

With the aim to reduce these potential problems, the use of encapsulated version (ENC) has been proposed. As the manufacturer pre-dose the powder and liquid inside a capsule, the powder-liquid ratio is standardized, and the mechanical mixing provides a more homogeneous material [14, 15]. There is a lack of information about the impact of different GIC supply forms on restoration survival. To the best of our knowledge, only one clinical trial compared different GIC presentations on occlusal restorations in permanent molars, with promising results for the encapsulated version [16].

Therefore, the aim of the present randomized clinical trial (RCT) is to compare the survival rate of primary molars restorations performed with hand-mixed and encapsulated versions of GIC after 24 months follow-up.

## Methods

This article was reported according to CONSORT (Consolidated Standards of Reporting Trial) [17] guidelines, and the checklist is available as a supplementary file.

### Trial design and ethical aspects

This is a two-sided equity, parallel arms, one-to-one allocation ratio, single-blinded (examiner), controlled randomized clinical trial. The present RCT is nested to a caries diagnosis RCT intitled CARies DEtection in Children 1 (CARDEC 01) [18].

It was conducted in dental office setting with children who sought dental care at the School of Dentistry, University of São Paulo, Brazil. This study was approved by the local research ethics committee (protocol #864.396) and registered on 15/10/2014 on the ClinicalTrials.gov platform (NCT02274142). All parents or legal guardians signed the informed consent form.

Initially children from three to six years of age would be included, however, to cover the largest number of children included in CARDEC 01 trial, we increased this age group to three to ten years old. This change is declared in the study registry. In addition, we have performed an Intention to Treat (ITT) and subgroup analysis that were not anticipated on trial registry.

### Sample size

The sample size estimation was performed on the Power and Sample Size website (http://powerandsamplesize.com/). A two-tailed hypothesis was considered. We considered parameters from a systematic review [19], which reported an average survival rate of 78% after 2 years of follow-up (mean for occlusoproximal and occlusal restorations after 2 years of follow-up). A clinically important difference of 15% on survival rate between ENC and H/M groups was considered. We added 20% for possible losses to follow up, and 20% for the cluster effect, as the same child could have more than one tooth included in the study. Thus, 116 teeth were needed per group, reaching a minimal sample size of 232 teeth. A significance level of 5% and a power of 80% were used for calculation.

### Eligibility criteria

Healthy children aged 3 to 10 years, who had sought dental treatment in the University of São Paulo and participating in CARDEC 01 study [18] were assessed. Bilateral bitewings were taken in all included participants in the diagnostic RCT [18]. Only children presenting dentin caries lesion in primary molars detected clinically as a cavitation or radiographically as dentin radiolucency in in occlusal and/or occlusoproximal surfaces were eligible to participate [20]. We included moderate or advanced lesions, with clinical and/or radiographic visualization of dentin involvement. However, when any signs or symptoms of irreversible pulp inflammation or pulp necrosis were detected clinically (nocturnal pain, fistula, abscess, pulp exposure, pathological mobility) or radiographically (radiolucency into the pulp, furcal bone radiolucency or pathological root resorption) the tooth was excluded.

In cases where there was any doubt about pulpal involvement, pathological root resorption, furcation bone lesions or any other problems, we have performed periapical radiographs. Children with severe behavioral problems and those whose parents or guardians refused to sign the informed consent form were excluded.

### Randomization, allocation concealment, and implementation

A sequence of random numbers, stratified according to caries experience and in blocks of four, was generated using a Random Allocation Software 2.0 [21], and these numbers were packed in opaque sealed envelopes by an external member of the research team who did not participate in the operative stages of the study to guarantee the allocation concealment.

Children with low experience were considered those who presented dmfs less than or equal to 3, and children with high experience those whose dmfs were higher than 3 [20].

The randomization implementation was made by two team members who did not participate in the research's operative phase, as they were responsible for the treatment plan design. Thus, the operators received the treatment plan, avoiding selection bias.

The randomization unit was the tooth, so each child could be able to contribute with more than one tooth to the study, and therefore could have received different restorative material in different teeth.

### Blinding

The restorations were performed with GIC in hand-mixed (H/M) and encapsulated (ENC) versions. Thus, there was no possibility of blinding participants and operators. Only the outcome assessors were blinded regarding groups.

### Interventions

All treatments were performed by trained general dentists, specialists, and graduate students in Pediatric Dentistry. The operators’ training was done through theoretical classes and meetings to solve any remaining questions before starting the clinical phase. In addition, one senior researcher supervised the treatments. If doubts related to the clinical protocols arose, the responsible researcher would clarify them, ensuring the operators follow the protocol. After clinical and radiographic evaluation and agreeing to participate, children were randomized to the following treatments groups:

- Hand-mixed Group: restorations performed with GIC Fuji IX Gold Label^®^ (GC Europe NV, Leuven, Belgium), in the hand-mixed version with manual dosage and handling.
- Encapsulated Group: restorations performed using GIC Equia Fill^®^ (Easy/Quick/Unique/Intelligent/Aesthetic)—GC Europe NV, Leuven, Belgium, in the pre-dosed encapsulated version and mechanical manipulation.

Materials’ composition information is depicted in Additional file 1.

### Restorative procedures

The procedures were performed without the use of local anesthesia. High speed rotary burs were used for enamel removal to gain access in case of non-cavitated dentin lesions detected radiographically.

Selective caries removal was performed in occlusal and occlusoproximal lesions for both groups. The caries dentin was removed using hand instruments appropriate for the cavity size. Cavity conditioner (polyacrylic acid—GC cavity conditioner) was applied for 15 s using a wet cotton pellet. Rinsing was performed using a sequence of three wet cotton pellets followed by three dry cotton pellets. The GIC was mixed and applied according to the following groups:

#### H/M

The GIC hand-mixed was handled in a paper block with a plastic spatula (GC Corporation, Japan) by two trained operators following the manufacturer’s recommendations. The GIC was inserted into the cavity with #1 spatula. Press finger was performed with a gloved finger coated with petroleum jelly for 10 s. After the initial material setting (from 3 to 5 min), the occlusion was checked with carbon paper and adjusted when necessary. Finally, restoration protection was performed with petroleum jelly.

#### ENC

The GIC (Equia Fill—GC Corporation, Japan) was activated, following the manufacturer's recommendations, and taken to the mixer by a team member, other than the operator of the restorative procedure. The encapsulated material was inserted directly from the capsule using a capsule applier (Riva Applicator—SDI Limited®, Australia). Press finger was performed with a gloved finger coated with petroleum jelly for 10 s. After the initial material setting (from 3 to 5 min), the occlusion was checked with carbon paper and adjusted when necessary. Restoration protection was performed with petroleum jelly.

For all occlusoproximal cavities, metal matrixes and wooden wedges were used. All subjects were instructed not to eat for one hour and received instructions on sugar consumption and oral hygiene for caries control.

### Outcome evaluation

The restorations were assessed clinically continuously, with a minimum of 4 months and a maximum of 8 months between evaluations, up to 24 months by two trained, calibrated, and blinded examiners (D.P.R. and L.B.C), following the Frencken and Holmgren [22] criteria for occlusal restorations and Roeleveld et al. [23] criteria for occlusoproximal restorations and the Additional files 2 and 3, respectively, shows this in more detail. Kappa test was performed to evaluate the level of inter-examiner agreement.

We considered as success for occlusal restorations the scores 0, 1 e 7, and for occlusoproximal, 00 and 10, which indicate the presence of good restoration, or only minor defect, with no repair needed. If other minor or major restoration failure was noted, the repair of the restoration was performed. In case of bulk fracture, the tooth received a new restoration.

For survival analysis, reintervention was not considered. If the primary restoration needed any repair or even replacement, we considered it a failure regarding the restorative procedure. All participants received full dental treatment, except orthodontic appliances. Parents could bring the child if any treatment need was detected between the pre-determined assessments.

### Outcomes

This study's primary outcome was restorations' survival performed with hand-mixed and encapsulated GIC in occlusal and occlusoproximal cavities in primary molars after 24 months follow-up. As a secondary outcome, we aimed to evaluate the cost-effectiveness of both GIC, considering the longevity of restorations. The secondary outcome will be published separately elsewhere.

### Statistical analysis

The analyses were performed in Stata 13 (StataCorp, USA). Kaplan–Meier's analysis shows the survival of the restorations over the 24 months of follow-up. Participants evaluated at least once during the study were included in the analysis.

To evaluate the primary outcome, we performed a survival analysis. Additionally, we performed an intention to treat (ITT) analysis, considering the success and failures at 24 months of follow-up.

To evaluate the association between restoration survival and independent variables such as surface (occlusal or occlusoproximal), caries experience (dmfs ≤ 3 or > 3), type of molar (first or second molar), sex (male or female), age and arch (upper or lower), Cox Regression with shared frailty was used. Initially, the analysis was performed in a univariate model. Independent variables reaching a *p*-value < 0.20 (cavities and tooth type) fitted in the adjusted model. The final model only included variables showing *p* ≤ 0.05. As only the independent variable surface reached this *p*-value, we have conducted a subgroup analysis, considering the survival of occlusal and occlusoproximal restorations. Hazard ratios (HR) and relative risk (RR) were calculated with 95% confidence interval (CI). The significance level was set at 5%.

## Results

**Fig. 1:**
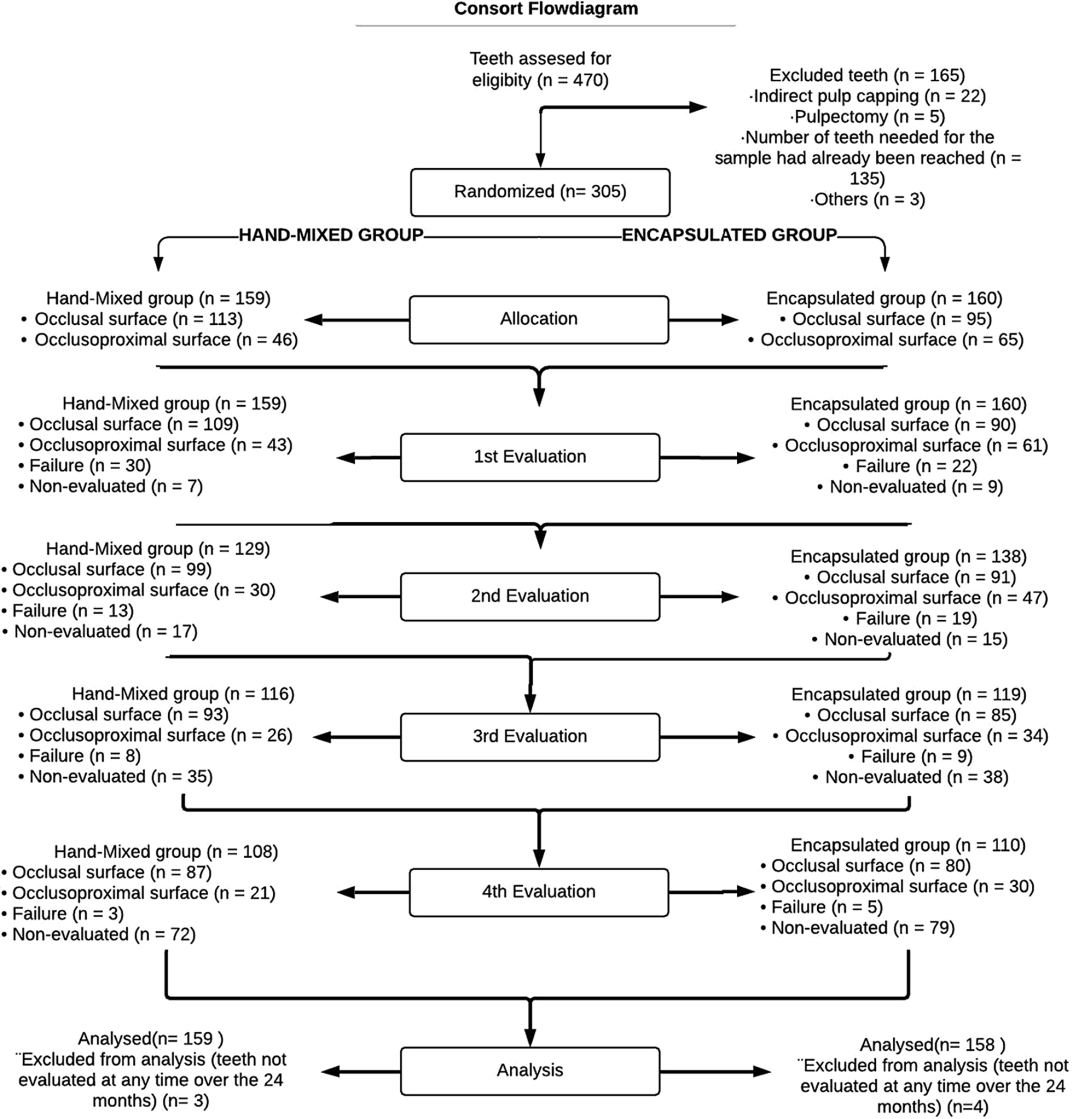
CONSORT flowchart of the participants’ progress through the trial phases

From 470 teeth in 147 children eligible to participate in the study, 305 were included. The reasons for exclusion of 165 teeth are described in Fig. 1. There was no case of pulp exposure during caries removal.

From 305 (323 cavities) teeth of 145 children included, 161 cavities were randomized for hand-mixed group and 162 cavities were allocated to the encapsulated group (Fig. 1). Considering all children, 67 (46.2%) were girls and 78 were (53.8%) boys. Moreover, 58 children (40.0%) were 3 to 4 years old and 87 (60.0%) were 5 to 10 years old, and 40 (27.6%) presented dmfs values from 0 to 3, and 105 (72.4%) presented dmfs of 4 or higher. The baseline characteristics are described in Table 1.

**Table 1:** Baseline characteristics of the participants, distribution according to the groups, and chi-square test results

Variables	Hand-mixed (n %)	Encapsulated (n %)	*p* value*
*Variables related to the children (323 teeth in 145 children)*			
**Sex**			0.111
Female	65 (44.2)	82 (55.8)	
Male	96 (54.6)	80 (45.4)	
**Age**			0.019
3 to 4 years-old	57 (41.3)	81 (58.7)	
5 to 10 years-old	104 (56.2)	81 (43.8)	
**dmfs**			0.720
0 to 3	35 (47.8)	38 (52.2)	
4 or more	126 (50.4)	124 (49.6)	
*Variables related to the teeth (n* = *323)*			
**Dental Arch**			0.958
Upper	83 (50.0)	83 (50.0)	
Lower	78 (49.7)	79 (50.3)	
**Type of Molar**			0.513
First molar	63 (47.7)	69 (52.3)	
Second molar	98 (51.1)	93 (48.7)	
**Surface**			0.030
Occlusal	113 (54.3)	95 (45.7)	
Occlusoproximal	48 (41.7)	67 (58.3)	
**Total**	161 (49.9)	162 (50.1)	

*Calculated by chi-square test adjusted by the cluster (children)

Moreover, 258 included teeth (positive follow-up rate of 79.9%) were followed-up up to 24 months, in 113 children (77.9%). The interexaminer Kappa value was 0.99. The drop-out for the hand-mixed group was 29 teeth (18.0%) and for the encapsulated group was 36 (22.2%), with a *p* value of 0.457 (by chi-square test adjusted by the cluster). Considering the 323 restorations included, only 7 (2.2%) samples were not assessed in any follow-up period.

**Fig. 2:**
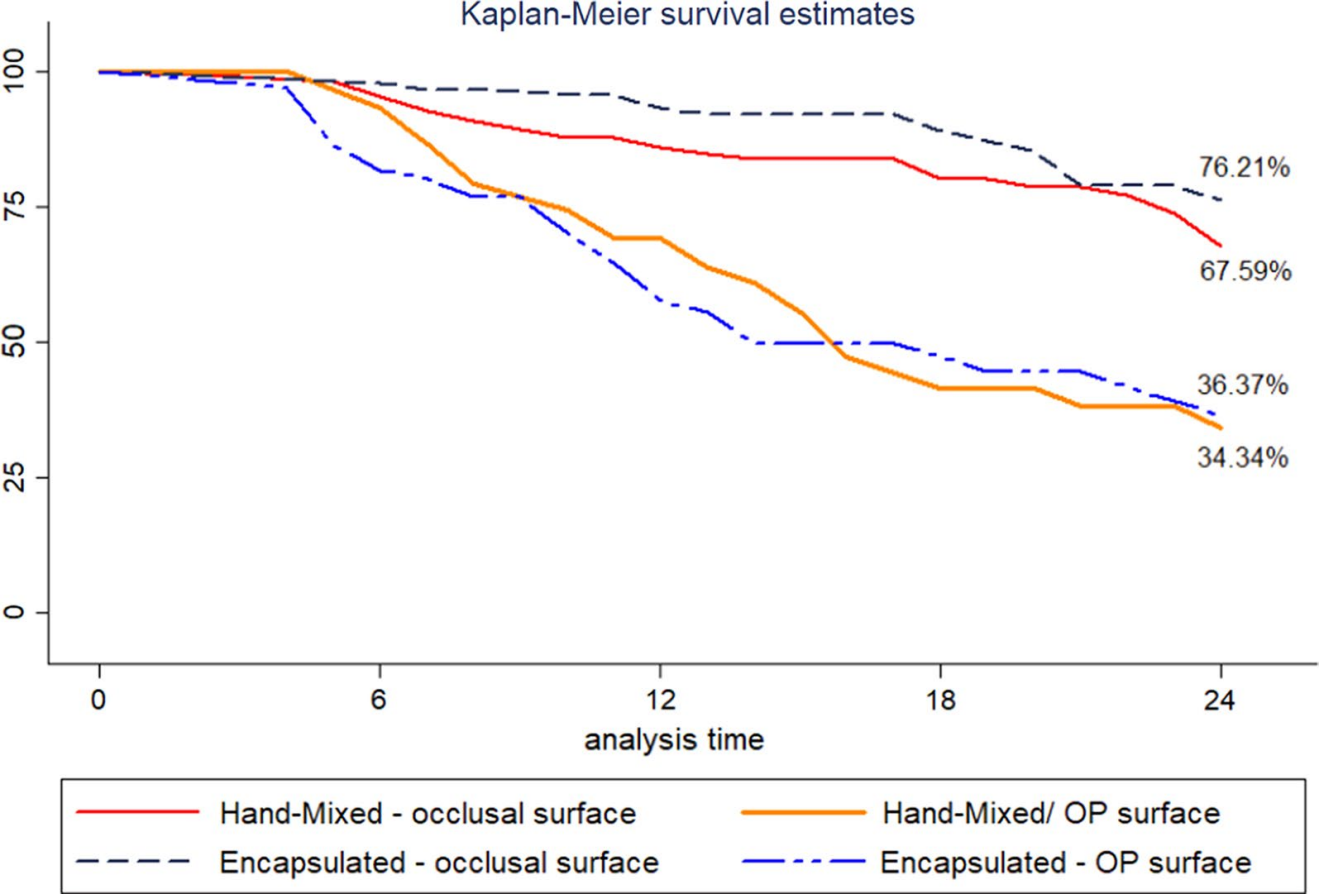
Kaplan–Meier survival estimates after 24 months follow-up

The Kaplan Meier curves show the estimated survival for the restorations according to restored surface after 24 months of follow-up (Fig. 2). Table 2 shows the most prevalent reasons of failure over the 24 months, according to Frencken and Holmgren (1999) [22] and Roeleveld et al. (2006) [23] criteria.

**Table 2:** Overall scores of restorations' success and failure

	Most prevalent scores of success and failure of restorations							
	1st evaluation		2nd evaluation		3rd evaluation		4th evaluation	
	Score	N (%)	Score	N (%)	Score	N (%)	Score	N (%)
Occlusal restorations—Frencken and Holmgren criteria	0	163 (80.29)	0	140 (80.46)	0	97 (76.99)	0	38 (65.52)
	1	11 (5.42)	1	4 (2.30)	1	1 (0.79)	1	2 (5.42)
	7	9 (4.43)	7	18 (10.34)	7	19 (15.08)	7	14 (4.43)
	Total evaluated	203	Total evaluated	174	Total evaluated	126	Total evaluated	58
Occlusoproximal restorations—Roeleveld et al. criteria	00	57 (54.80)	00	35 (54.69)	00	22 (55)	00	8 (57.14)
	10	14 (13.46)	10	9 (14.06)	10	11 (27.5)	10	2 (14.3)
	30	19 (18.27)	30	11 (17.18)	30	5 (12.5)	30	3 (21.43)
	Total evaluated	104	Total evaluated	64	Total evaluated	40	Total evaluated	14

Tables 3, 4 and 5 shows the ITT results, Cox Regression with shared fragility and subgroup analysis, respectively, and no statistically significant differences were found. Table 6 shows the values of Annual Failure Rate, calculated according to the intention-to-treat analysis (ITT).

**Table 3:** Survival analysis considering the primary outcome and ITT analysis for 24 months of follow-up

Trial groups	Survival proportion %	SE	HR (95%CI)	*p*
*Survival analysis (primary outcome)*				
Hand-mixed group	60.9	0.04	1.00	0.626*
Encapsulated group	59.3	0.05	0.91 (0.61 to 1.35)	

Trial groups	Success	Failure	RR (95%CI)	*p*
	N (%)	N (%)		
*Success at 24 months (intention to treat analysis)*				
Hand-mixed group	100 (62.1)	61 (37.8)	1.00	0.498**
Encapsulated group	99 (61.1)	63 (38.9)	0.88 (0.62 to 1.26)	

SE = Standard Error; HR = Hazard ratio; 95%CI = 95% confidence interval; RR = relative risk

*p value calculated by Cox regression with shared frailty

**p value calculated by ITT

**Table 4:** Cox Regression with shared frailty in univariate and adjusted models -analysis between failures in restorations and associated factors

	Unadjusted HR (95%CI)	*p*	Adjusted HR (95%CI)	*p*
*Group (ref.: hand-mixed)*				
Encapsulated	0.99 (0.66 to 1.48)	0.967	0.90 (0.60 to 1.35)	0.617
*Sex (ref.: female)*			*	
Male	0.86 (0.56 to 1.34)	0.516		
*Age (ref.: 3 to 4 years-old*			*	
5 to 6 years-old	1.27 (0.81 to 1.98)	0.294		
dmfs (ref.: 0 to 3)			*	
4 or more	0.84 (0.51 to 1.39)	0.500		
*Dental arch (ref.: upper)*			*	
Lower	0.97 (0.65 to 1.45)	0.894		
*Molar type (ref.: 1st molar)*				
2nd molar	0.55 (0.37 to 0.82)	0.003	0.89 (0.59 to 1.34)	0.572
*Surface (ref.: Occlusal)*				
Occlusoproximal	3.97 (2.68 to 5.87)	< 0.001	3.83 (2.44 to 6.00)	< 0.001

HR = hazard ratio; 95% CI = 95% confidence interval

*Variable not included in the adjusted model

**Table 5:** Subgroup analysis considering occlusal and occlusoproximal restorations considering the survival of restorations performed with hand-mixed or encapsulated glass ionomer cement

Trial groups	Survival proportion %	SE	HR (95%CI)	*p*
*Occlusal restorations*				
Hand-mixed group	71.4	0.05	1.00	0.281
Encapsulated group	77.6	0.05	0.70 (0.36 to 1.35)	
*Occlusoproximal restorations*				
Hand-mixed group	34.3	0.08	1.00	0.717
Encapsulated group	30.2	0.07	1.10 (0.66 to 1.81)	

SE = Standard Error; HR = Hazard ratio; 95%CI = 95% confidence interval

*p value calculated by Cox regression with shared frailty adjusted by type of restoration (Occlusal or Occlusoproximal)

**Table 6:** Annual Failure Rate (AFR) according to intention-to-treat analysis

Trial group	Occlusal (%)	Occlusoproximal (%)
Hand-mixed	14.3	32.9
Encapsulated	11.2	34.9

AFR was calculated according to the formula (1 − y).z = (1 − x), in which “y” expresses the mean AFR and “x” the total failure rate at “z” years

### Harms

No damage or harms has been found in our trial. No pulp exposure occurred during treatments.

## Discussion

This RCT was performed in a controlled clinical environment (dental office setting) and demonstrated that both hand-mixed and encapsulated versions did not influence the restorations' survival. It is possible to observe a trend to better results for encapsulated material, especially for occlusal surfaces.

The GICs showed an excellent survival rate for occlusal cavities, corroborating with previous studies [19, 24]. This result was predictable because occlusal cavities usually present a more robust dental structure to support the restorative materials [25], favoring their longevity. For this reason, the occlusal cavities were considered in this study so that we could observe if there would be any difference in the clinical results obtained for the encapsulated material.

Table 1 shows an imbalance between the cavity type in the two groups, with more occlusal cavities in both groups. This fact led us to perform other analyses, which were not foreseen in the study design and registry, such as intention to treat (ITT) and subgroup analysis. These analyses, to our knowledge, make the results clearer, showing that despite the imbalance found in the baseline, there was no statistically significant difference in either group.

We have chosen to use petroleum jelly as a surface protector in this trial to balance both groups, excluding any confounding factor related to this topic, for example, using resin-based surface protection only for the Equia Fil® group. To our knowledge, there is only one RCT that compared GIC Hand-Mixed and Encapsulated, and the authors classified the used GIC as “medium viscosity” in permanent molar occlusal cavities, with results of one-year follow-up [16]. This study shows a higher success rate of encapsulated GIC, different from our findings. Some factors may have influenced the results such as the restorations performed on permanent molars, whose masticatory force is more potent than that applied to primary teeth, or factors such as type of tooth and location, operator, secondary lesions, individual risk of caries and bruxism which may have exacerbated the difference between the materials [26, 27]. Another relevant point may be related to manipulating the material, mainly for the hand mixed. In the present study, handling was performed by two operators with significant experience in teaching and handling these materials, which may explain the lack of difference between groups. We also hypothesize that as the RCT used medium-viscosity GIC, the material could be more sensitive to changes in dosage and handling.

Regarding Freitas et al. [16] study, another difference was found in the number of operators for the restorative procedures. This RCT had a team of five operators, while in the study of Freitas et al., restorations were performed by a single operator. We believe that this factor did not influence our results, as all operators received theoretical training through classes and meetings to clarify possible doubts related to the protocols used in this RCT. In addition, the researchers responsible for the research and who trained the operators were present in the clinical setting, and whenever there were questions related to the clinical protocols, they were immediately answered by those responsible for the RCT.

The present RCT was nested within another diagnostic trial, both with robust samples, requiring a big team, especially in the clinical phase. We had five trained and calibrated operators, three experienced professionals that had affinity to pediatric dentistry (but no specialization course), two graduate students and one specialization student in pediatric dentistry from the School of Dentistry, University of São Paulo. The operators’ experience may play a role in the restorations’ clinical performance. In our case, we believe that there was no influence, as they all received the same training and calibration exercises, and all treatments were supervised by one senior researcher.

Our results show that both materials showed similar survival rates in occlusoproximal cavities and had lower survival rates than occlusal restorations. However, this difference is not related to supply form but rather to issues related to the configuration of cavities [24, 28]. The reduced performance found for the occlusoproximal cavities might be related to the configuration of the cavities. As previously stated, GIC requires support from the surrounding structures [25], so variables related to cavity conformation need to be better studied for understanding the behavior of this restorative material in this type of cavity.

Another relevant issue related to occlusoproximal cavities might be humidity control. Lesions at the gingival or subgingival margin level are difficult for controlling the humidity and may negatively influence the restorative materials' clinical success. Such aspects compromise the survival of occlusoproximal restorations [24].

Besides the points that may be critical to the success of restorative treatments, especially regarding occlusoproximal cavities, we also have the points that should be highlighted to be successful in the restorative treatment of occlusal and occlusoproximal cavities of primary teeth. Points that need to be considered are good isolation of the operative field, remaining tooth structure, configuration/conformation of the cavity. These are points that we consider essential to be evaluated to achieve a good prognosis and predictability of the success or failure of the restorative treatment.

Our hypothesis that the restorations' survival of the ENC group for the different cavity types would be greater than the H/M group. A possible explanation is that there would be less influence of operators for dosage and handling, thus reducing the incorporation of air bubbles or the possible change in the powder-liquid ratio recommended by manufacturers [12–15]. As the dosage and handling in our H/M group were performed exclusively by two trained and experienced operators in GIC, it might have an impact on our results and must be considered a potential limitation on the generalizability of this trial’s results. Future studies might consider a pragmatic trial design or a “real-world” design, with less control over variables, to strengthen the scientific evidence on this topic and check if our previous assumptions were correct.

## Conclusion

The survival rate in primary molars is not influenced by the different supply forms of GIC. Also, occlusoproximal restorations present reduced performances when compared to occlusal cavities.

## Supplementary Information

**Additional file 1.** Composition of the restorative materials

**Additional file 2.** Frencken and Holmgren Criteria for occlusal restorations

**Additional file 3.** Roeleveld et al. criteria for occlusoproximal restorations

## Data Availability

The datasets generated and/or analyzed during the current study are not publicly available because there are a higher number of data that have not yet been evaluated/analyzed by the authors of the paper and that make up the secondary outcomes of this trial but are available from the corresponding author on reasonable request.

## Abbreviations

GIC: Glass ionomer cement
H/M: Hand-mixed
ENC: Encapsulated
ITT: Intention to treat
CONSORT: Consolidated Standards of Reporting Trials
CARDEC: CAries DEtection in Children
