# Supplementary material for: Survival rate of primary molar restorations is not influenced by hand mixed or encapsulated GIC: 24 months RCT
Source: BMC Oral Health. 2021 Jul 23;21:371. doi: 10.1186/s12903-021-01710-0 (PMC8305516; doi:10.1186/s12903-021-01710-0)
Supplement: Supplementary file 1 — Additional file 1. Composition of the restorative materials [file 12903_2021_1710_MOESM1_ESM.docx]

Additional File 1 - Composition of the restorative materials

| **Groups** | **Restorative Material** | **Manufacturer** | **Composition** |
| --- | --- | --- | --- |
| **Hand Mixed** | **Fuji IX**  **Gold Label^®^** | GC Corp | Powder: fluoroaluminosilicate glass, polyacrilyc acid powder |
|  |  |  | Liquid: polyacrilyc acid, polybasic carboxilic acid |
| **Encapsulated** | **Equia Fil**^®^ |  | Powder: 95% strontium fluoralumino-silicate, 5% polyacrilyc acid |
|  |  |  | Liquid: 40% aqueous polyacrilyc acid |
