# Supplementary material for: Survival rate of primary molar restorations is not influenced by hand mixed or encapsulated GIC: 24 months RCT
Source: BMC Oral Health. 2021 Jul 23;21:371. doi: 10.1186/s12903-021-01710-0 (PMC8305516; doi:10.1186/s12903-021-01710-0)
Supplement: Supplementary file 2 — Additional file 2. Frencken and Holmgren Criteria for occlusal restorations [file 12903_2021_1710_MOESM2_ESM.docx]

Additional File 2 - Frencken and Holmgren Criteria for occlusal restorations

| **Score** | **Criteria** |
| --- | --- |
| 0 | Present, good |
| 1 | Present, slight marginal deffect for whatever reason, at any one place wich is less than 0.5mm in depth. No repair is needed |
| 2 | Present, marginal defefct for whatever reason, at any one place which is deeper than 0.5mm but less than 1.0mm. Repair is needed |
| 3 | Present, gross defect of more than 1.0mm in depth. Repair is needed |
| 4 | Not present, restoration has (almost) completely disappeared. Treatment is needed |
| 5 | Not present, other restorative tretatment has been performed |
| 6 | Not present, tooth has been extracted |
| 7 | Present, wear and tear gradually over larger parts of the restoration but are less than 0.5mm at the deepest point. No repair is needed |
| 8 | Present, wear and tear gradually over larger parts of the restoration which are deeper than 0.5mm. Repair is needed |
| 9 | Unable to diagnose |

Note: Restorations considered to have survived are scored by codes: 0, 1 or 7; those considered to have failed by codes: 2, 3, 4 and 8; while those considered to be unrelated to success and failure are coded: 5 and 6.
