# Supplementary material for: Survival rate of primary molar restorations is not influenced by hand mixed or encapsulated GIC: 24 months RCT
Source: BMC Oral Health. 2021 Jul 23;21:371. doi: 10.1186/s12903-021-01710-0 (PMC8305516; doi:10.1186/s12903-021-01710-0)
Supplement: Supplementary file 3 — Additional file 3. Roeleveld et al. criteria for occlusoproximal restorations [file 12903_2021_1710_MOESM3_ESM.docx]

Additional File 3 - Roeleveld et al. criteria for occlusoproximal restorations

| **Score** | **Criteria** |
| --- | --- |
| 00 | Restoration still present, correct |
| 10 | Restoration present, slight defect at the margin and/or wear of the surface; <0.5mm in depth, no reparation needed |
| 11 | Restoration present, defect at the margin and/or wear of the surface; >0.5mm in depth, repair needed |
| 12 | Restoration presente; underfilled > 0.5mm, no gap, repair needed |
| 13 | Restoration overfilled > 0.5mm, repair needed |
| 20 | Secondary caries, discoloration in depth, surface hard and intact, caries within dentin; repair needed |
| 21 | Secondary caries. Surface defect., caries within dentin; repair needed |
| 30 | Restoration not presente, bulk fracture, loose, (partly) lost; repair needed (if still possible without exposing the pulp) |
| 40 | Inflamation of the pulp (restoration still in situ, not categorized in the former categories); fistula os severe pain complaints; extraction needed |
| 50 | Tooth not present because of extraction |
| 60 | Tooth not present because of shedding |
| 70 | Tooth nor present because of extraction or shedding; unable to diagnose |
| 90 | Patient not present |

Note: Restorations considered to have survived are scored by codes: 00 and 10; those considered to have failed by codes: 11, 12, 13, 20, 21, 30 or 40; while those considered to be unrelated to success and failure are coded: 50, 60, 70 or 90.
